# Cross-Sectional Blood Metabolite Markers of Hypertension: A Multicohort Analysis of 44,306 Individuals from the COnsortium of METabolomics Studies

**DOI:** 10.3390/metabo12070601

**Published:** 2022-06-28

**Authors:** Panayiotis Louca, Ana Nogal, Aurélie Moskal, Neil J. Goulding, Martin J. Shipley, Taryn Alkis, Joni V. Lindbohm, Jie Hu, Domagoj Kifer, Ni Wang, Bo Chawes, Kathryn M. Rexrode, Yoav Ben-Shlomo, Mika Kivimaki, Rachel A. Murphy, Bing Yu, Marc J. Gunter, Karsten Suhre, Deborah A. Lawlor, Massimo Mangino, Cristina Menni

**Affiliations:** 1 Department of Twin Research, King’s College London, London SE1 7EH, UK; panayiotis.louca@kcl.ac.uk (P.L.); ana.nogal_macho@kcl.ac.uk (A.N.); massimo.mangino@kcl.ac.uk (M.M.); 2 Nutrition and Metabolism Section, International Agency for Research on Cancer, 69372 Lyon, France; aurelie.moskal@inserm.fr (A.M.); gunterm@iarc.fr (M.J.G.); 3 Population Health Sciences, Bristol Medical School, University of Bristol, Bristol BS8 2BN, UK; n.goulding@bristol.ac.uk (N.J.G.); y.ben-shlomo@bristol.ac.uk (Y.B.-S.); d.a.lawlor@bristol.ac.uk (D.A.L.); 4 MRC Integrative Epidemiology Unit, University of Bristol, Bristol BS8 2BN, UK; 5 Department Epidemiology and Public Health, University College London, London WC1E 7HB, UK; martin.shipley@ucl.ac.uk (M.J.S.); joni.lindbohm@helsinki.fi (J.V.L.); m.kivimaki@ucl.ac.uk (M.K.); 6 Department of Epidemiology, Human Genetics and Environmental Sciences, School of Public Health, The University of Texas Health Science Center, Houston, TX 77030, USA; taryn.alkis@uth.tmc.edu (T.A.); bing.yu@uth.tmc.edu (B.Y.); 7 Clinicum, Department of Public Health, University of Helsinki, P.O. Box 20 Helsinki, Finland; 8 Division of Women’s Health, Brigham and Women’s Hospital and Harvard Medical School, Boston, MA 02115, USA; jhu13@bwh.harvard.edu (J.H.); krexrode@bwh.harvard.edu (K.M.R.); 9 Faculty of Pharmacy and Biochemistry, University of Zagreb, 10000 Zagreb, Croatia; dkifer@pharma.hr; 10 Copenhagen Prospective Studies on Asthma in Childhood, Herlev and Gentofte Hospital, University of Copenhagen, 2820 Gentofte, Denmark; ni.wang@dbac.dk (N.W.); chawes@copsac.com (B.C.); 11 Department of Biotechnology and Biomedicine, Technical University of Denmark, 2800 Kongens Lyngby, Denmark; 12 NIHR Applied Research Collaboration West, University Hospitals Bristol and Weston National Health Service Foundation Trust, Bristol BS1 2NT, UK; 13 School of Population and Public Health, University of British Columbia, Vancouver, BC V6T 1Z3, Canada; rachel.murphy@ubc.ca; 14 Cancer Control Research, BC Cancer, Vancouver, BC V5Z 1G1, Canada; 15 Department of Biophysics and Physiology, Weill Cornell Medicine-Qatar, Doha 24144, Qatar; karsten@suhre.fr; 16 Bristol NIHR Biomedical Research Centre, University of Bristol, Bristol BS1 2NT, UK; 17 NIHR Biomedical Research Centre at Guy’s and St Thomas’ Foundation Trust, London SE1 9RT, UK

**Keywords:** metabolomics, hypertension

## Abstract

Hypertension is the main modifiable risk factor for cardiovascular morbidity and mortality but discovering molecular mechanisms for targeted treatment has been challenging. Here we investigate associations of blood metabolite markers with hypertension by integrating data from nine intercontinental cohorts from the COnsortium of METabolomics Studies. We included 44,306 individuals with circulating metabolites (up to 813). Metabolites were aligned and inverse normalised to allow intra-platform comparison. Logistic models adjusting for covariates were performed in each cohort and results were combined using random-effect inverse-variance meta-analyses adjusting for multiple testing. We further conducted canonical pathway analysis to investigate the pathways underlying the hypertension-associated metabolites. In 12,479 hypertensive cases and 31,827 controls without renal impairment, we identified 38 metabolites, associated with hypertension after adjusting for age, sex, body mass index, ethnicity, and multiple testing. Of these, 32 metabolite associations, predominantly lipid (steroids and fatty acyls) and organic acids (amino-, hydroxy-, and keto-acids) remained after further adjusting for comorbidities and dietary intake. Among the identified metabolites, 5 were novel, including 2 bile acids, 2 glycerophospholipids, and ketoleucine. Pathway analysis further implicates the role of the amino-acids, serine/glycine, and bile acids in hypertension regulation. In the largest cross-sectional hypertension-metabolomics study to date, we identify 32 circulating metabolites (of which 5 novel and 27 confirmed) that are potentially actionable targets for intervention. Further in-vivo studies are needed to identify their specific role in the aetiology or progression of hypertension.

## 1. Introduction

High blood pressure (BP) is the leading modifiable risk factor for cardiovascular disease (CVD), global morbidity, and mortality, and recognised as the greatest single risk factor contributing to the global burden of disease—responsible for 8.5 million deaths per year [1,2]. BP is complex and multifactorial by nature, influenced by genomic, lifestyle, and environmental factors and a multitude of physiological pathways [3,4]. Reducing the burden of hypertension is therefore important, and can be achieved via improving the coverage of treatment and primary prevention, which has become an objective of many global and national health initiatives [2]. However, the pace of progress in this regard has stalled [5], partly due to the deceleration of research investigating novel targets, which could then be used for drug/treatment development [6].

Circulating metabolites comprise the intermediate and end-products of metabolic pathways that reflect physiological processes [7]. Therefore, metabolites are well suited to characterise the influence of key measures of disease risk brought about by environmental, nutritional, or lifestyle factors, thus leading to the discovery of novel metabolic aspects of complex diseases [8,9].

Previous work has identified numerous metabolites linked with hypertension and blood pressure [4,10,11,12,13]. The majority of which falling into the lipid and amino acid classes [4]. This includes the dicarboxylic fatty acid hexadecanedioate that is associated with increased BP mortality [11] and heart failure [14], and the amino acid phenylacetylglutamine [15].

Though metabolomics has been promising in identifying disease biomarkers, assays are expensive, typically performed on <1000 subjects, and no single metabolomics platform covers the full range of detectable metabolites [16], with a modest overlap between platforms [16]. Accordingly, studies on the metabolic mechanisms underlying hypertension are limited, and lack sufficient sample size [16]. The COnsortium of METabolomics Studies (COMETS) is a partnership between many well-characterised prospective cohort studies to provide a framework for collaborative metabolomics research, specifically initiated to advance our understanding of the metabolomes’ involvement in disease aetiology, diagnosis, and treatment, by overcoming some of the abovementioned challenges [16].

The aim of this study is to identify blood metabolite markers of essential hypertension, which may provide novel actionable targets and pathways for hypertension treatments. To achieve this, we will conduct a metabolome-wide association study (MWAS) of hypertension by integrating data from nine intercontinental cohorts, including 44,306 individuals of different ethnicities, sex, age, and metabolomic platforms, from COMETS. We then investigate canonical pathways underlying the hypertension-associated metabolites.

## 2. Results

### 2.1. Study Demographics

We included 12,479 essential hypertensive cases and 31,827 non-hypertensive controls from 9 cohorts (*n* = 44,306) and analysed a total of 813 serum/plasma metabolites. The demographic characteristics of the study sample are presented in Table 1. Average age ranged from 27.95 (±5.69) to 74.67 (±2.84) years, and on average subjects were overweight with an average body mass index (BMI) in the range of 26.1 (±4.81) to 28.93 (±5.93) kg/m^2^. In total we included 24,864 female and 19,442 male individuals, of which 19,448 were of White/European ancestry, 2631 of black ancestry, 4163 of Asian or Hispanic ancestry, and 74 of other ancestries.

### 2.2. Discovery Analysis

**Figure 1:**
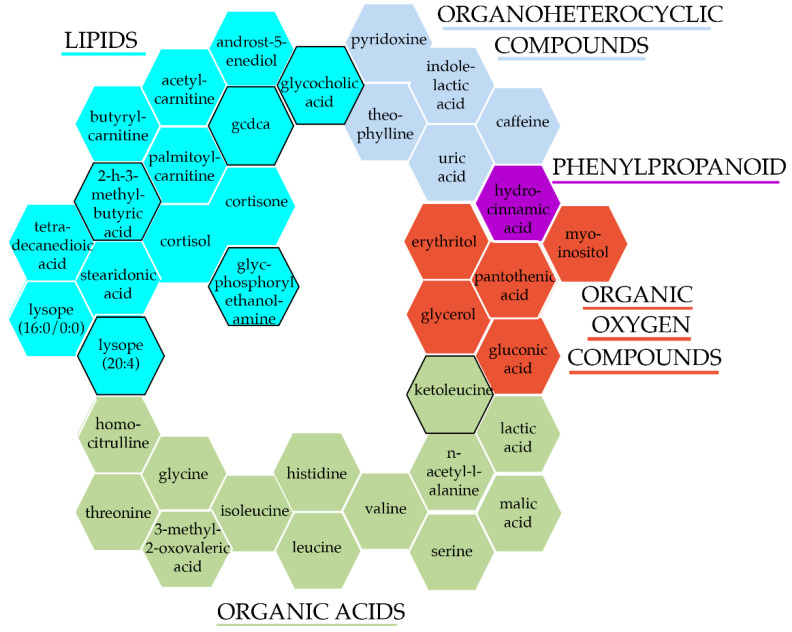
Hypertension-associated metabolites after adjusting for traditional risk factors. Colours and groupings represent each metabolite super class, while a black border signifies novel-associations with hypertension/BP.

To identify circulating metabolites associated with essential hypertension in the overall sample, we meta-analysed the age, age^2^, sex, BMI, and ethnicity adjusted results from each of the nine contributing cohorts. We identified 38 metabolites associated with hypertension cross-sectionally, after adjusting for multiple testing (Figure 1 and Supplementary Table S2). To the best of our knowledge, 6 of these were novel hypertension associations. These 38 hypertension-associated metabolites were comprised of 14 lipids (36.8%) (6 fatty acyls, 5 steroids, and 3 glycerophospholipids), 13 organic acids (34.2%) (9 amino acids and peptides, 2 short-chain-keto acids, and 2 hydroxy acid), 5 organic oxygen compounds (13.2%) (3 carbohydrates and 2 alcohols), 5 organoheterocyclic compounds (13.2%) (3 purines, 1 indole, and 1 pyridoxine), and 1 (2.6%) phenylpropanoid (hydrocinnamic acid) (Figure 1)**.**

For the 38 metabolites that passed our multiple testing threshold, heterogeneity statistics ranged from 0% to 65% (median = 26.6%) for I^2^, and from 0 to 14 (median = 1.1) for Cochrane’s Q.

**Figure 2:**
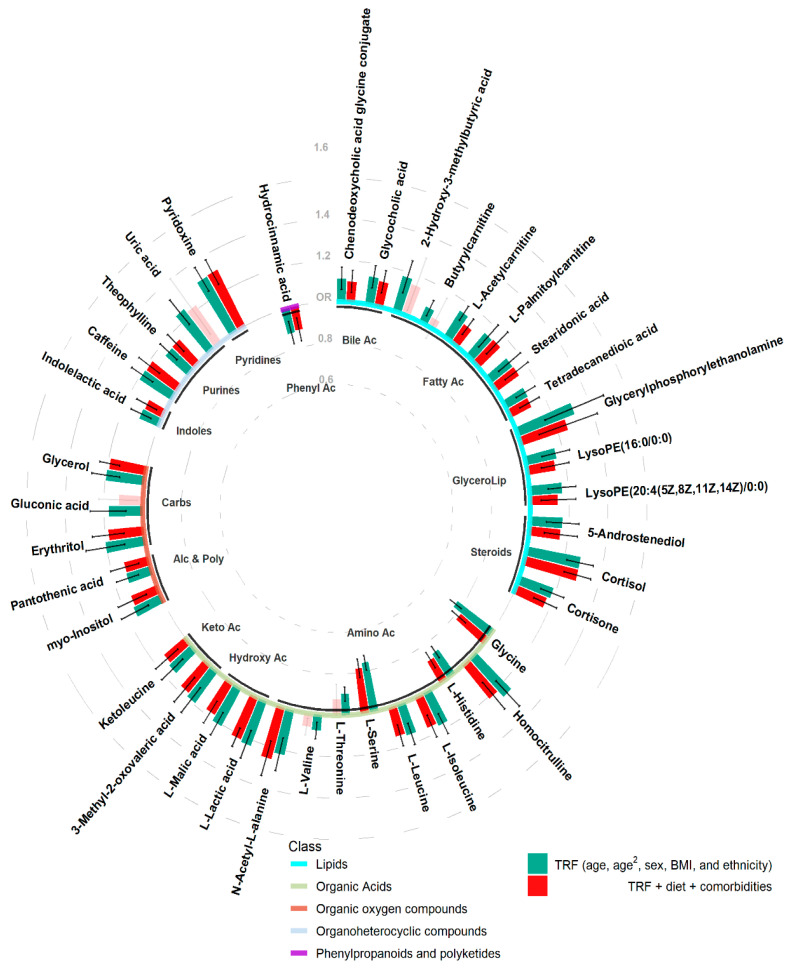
Odds ratio’s and 95% confidence intervals of hypertension associated metabolites adjusting for traditional risk factors (TRF) (green) or TRF + diet + comorbidities (red). Semi-transparent bars illustrate analyses not passing the pre-defined alpha threshold. Base line colours represent the super class of metabolites, while sub-base labels indicate the metabolite sub class.

Of the 14 lipids, all were positively associated with hypertension, and the strongest association was Glycerylphosphorylethanolamine (odds ratio (OR) [95%CI] = 1.31 [1.16, 1.48]). The strongest positive and negative associations of the 13 organic acids, was Homocitrulline (OR [95%CI] = 1.25 [1.17, 1.34]) and Serine (OR [95%CI] = 0.76 [0.72, 0.8]), respectively. Of 5 positively associated organic oxygen compounds, erythritol elicited the strongest association (OR [95%CI] = 1.2 [1.11, 1.31]). Similarly, all 5 organoheterocyclic were positively associated with hypertension, with pyridoxine (OR [95%CI] = 1.31 [1.24, 1.37]) having the highest OR. While the only phenylpropanoid to be identified, hydrocinnamic acid, was negatively associated with hypertension (OR [95%CI] = 0.88 [0.82, 0.94]) (Figure 2).

### 2.3. Sensitivity Analysis

To investigate the robustness of these associations, using inverse-variance meta-analysis we further pooled the fully adjusted model (further adjusting for dietary salt and alcohol intakes and comorbidities) estimates for the 38 hypertension-associated metabolites.

Out of the 38 metabolites, 32 remained significant after further adjustments and multiple testing (*p* < 0.05/38 = 1.3 × 10^−3^).

The more robust hypertension-associated metabolites were still predominantly lipids (12 of 14, 37.5%) and organic acids (11 of 13, 34.4%), but also 4 of the 5 organic oxygen compounds and 4 of the 5 organoheterocyclic compounds remained, as did hydrocinnamic acid. Of the 32 metabolites, 5 of the novel hypertension-associations also remained associated (Figure 2 and Supplementary Table S2).

While further accounting for dietary influences and comorbidities, the organoheterocyclic metabolite, pyridoxine still elicited the strongest positive effect on hypertension (OR [95%CI] = 1.31 [1.24, 1.38]), while the amino acid serine also was the strongest negative association (OR [95%CI] = 0.78 [0.74, 0.83]) (Figure 2).

### 2.4. Stratified Analyses

To investigate if key demographic factors may be driving metabolite associations, we conducted a sub-analysis by stratifying our samples by (i) sex and (ii) race/ethnicity. We then pooled the estimates for both the traditional-risk factor adjusted and fully adjusted models for the 32 hypertension associated metabolites.

**Figure 3:**
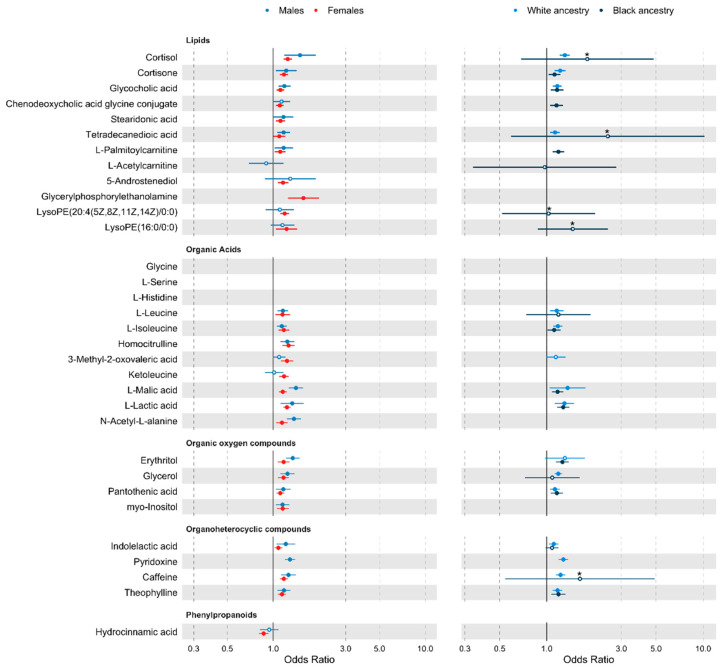
Forest plot of traditional risk factor adjusted analyses stratified by sex, and ethnicity. Metabolites are grouped by class. Points represents odds ratio (OR), and tails show the 95% confidence interval (CI). For an improved scale, associations with an upper CI > 3 have been truncated, reducing the standard error by 2.5 times, and are identified by a * above the OR point. For true values see Supplementary Figure S2. Metabolites not passing an α level of 0.05 are shown with a white point. Metabolites where there were fewer than 2 cohorts with the metabolite detected in >80% of the stratified sample are missing.

For the traditional risk factor adjusted estimates, when stratifying by sex, out of the 32 metabolites, 20 metabolites were associated in the male only sample, and 27 were associated in the female only sample. When we stratified by race/ethnicity 14 of the 32 metabolites were associated with hypertension in those of White/European ancestry, and 10 in those of Black ancestry (Figure 3).

Results when further adjusting for comorbidities and alcohol and salt intakes remained consistent. For the fully adjusted model estimates, 18 of the 20 metabolites remained associated in males, while all 27 hypertension-associated metabolites remained significant in females (at *p* < 0.05). For race/ethnicity, 15 metabolites were associated in White/European individuals and all 10 previously identified metabolites were associated in those of Black ancestry (at *p* < 0.05) (Supplementary Figure S2).

### 2.5. Pathway Analysis

We further conducted canonical pathway analysis of the 32 hypertension-associated metabolites using the IPA database to explore the metabolic footprint of those metabolites. Canonical pathway analysis suggests that the probe set of metabolites were likely to be involved in tRNA charging, glycine, leucine and serine pathways, folate pathways, and bile acid biosynthesis (Supplementary Figure S3).

## 3. Discussion

In the largest MWAS of essential hypertension study to date, including over 44,000 individuals from COMETS, we identify 32 circulating metabolites associated with hypertension, after adjusting for traditional risk factors, diet, comorbidities, and multiple testing. Five of these metabolites were novel and had not yet been previously shown to associate with hypertension or blood pressure. These include 4 lipids (2 Glycerophospholipids, and 2 bile acids) and 1 organic acid (the short-chain keto acid, ketoleucine). The majority of the identified metabolites were lipids, including steroids (2) and bile acids (2), and organic acids, including amino acids (7). Organic oxygen compounds (4), organoheterocyclic compounds (4), and phenylpropanoids (1) were also identified. Pathway analysis in the IPA database support these findings, identifying tRNA charging, as the pathway in which the metabolite probe set was most likely to be involved, and additionally showing significant overlap with glycine and serine pathways (Supplementary Figure S3). Pathway analysis also suggests a renal role via bile acids and folate.

Results in women and men were mostly statistically consistent with each other, as were those comparing White/European to African origin ethnicity. However, for malic acid the confidence intervals between males and females did not overlap, with an OR [95%CI] in men of 1.41 [1.26, 1.57] and 1.15 [1.09, 1.23] in women, despite these estimates being imprecise.

We find novel associations between circulating levels of the secondary bile acids glycocholic acid and chenodeoxycholic acid glycine conjugate (GCDCA), and hypertension, both of which were positively associated. Results were consistent when stratifying by sex and by race/ethnicity. Bile acids are recognised to facilitate transport of dietary lipids and fat-soluble vitamins; regulate glucose homeostasis, lipids and lipoprotein metabolism, energy expenditure; and influence inflammation [17]. In addition to these, the vasoactive properties of bile acids have been recognised for decades, although the mechanisms by which they act has not been defined [18]. The expression of bile acid receptors in endothelium has conjured hypotheses relating to nitric oxide production, a potent vasodilator [18]. In murine models of hypertension the infusion of bile acids (deoxycholic acid) significantly reduced arterial BP by 12 mmHg [19]. Although glycocholic acid has not previously been associated with hypertension or blood pressure, previous evidence suggests links with other cardiovascular factors, such as atrial fibrillation [20], and its precursor, cholic acid, has been causally linked with the development of hypertension within murine models [21]. GCDCA on the other hand has been linked with toxicity in cardiac mitochondria in vitro [22].

Amino acids, including serine, glycine, histidine, and alanine, have previously and consistently been implicated in a variety of cardiometabolic traits, including hypertension and CVD [7,11,23], and amino-acid metabolism is considered one of the critical pathways involved in hypertension regulation [24]. Indeed, amino acids have been implicated in intracellular signalling, whereby dysregulation of amino acid metabolism may result in inflammation, oxidative stress, and insulin resistance, states involved in the aetiology of hypertension [25]. Essential amino acids cannot be synthesised de novo and must be acquired from dietary sources. Here we report negative associations between the essential amino acid histidine and hypertension, and positive associations for the essential amino acids isoleucine, and leucine, in accordance with previous studies on coronary heart disease [26]. For the non-essential amino acids, both glycine, and serine were negatively associated with hypertension. Abundance of non-essential amino acids are symbolic of protein catabolism and links have been made to their involvement in pulsatile haemodynamics [23]. These findings are in line with previous reports [7,11,23]. We have previously reported inverse associations between pulse wave velocity and various amino acids in a sample of 1797 normotensive White/European women [7]. Urinary levels of amino acids have been repeatedly correlated with blood pressure, hypertension, and vascular factors. Several amino acids, including, serine, glycine, and histidine, were inversely related to SBP and pulse pressure in individuals of Black ancestry [23]. On the other hand, of the seven hypertension-associated amino acids we find here, only isoleucine, remains significant when stratifying by Black ancestry. These findings are supported by our canonical pathway analysis in the IPA database, which also matches the findings of Zhao and colleagues [24].

There is a long-standing relationship between various lipids and CVD. Here, we identify 14 lipids positively associated with hypertension (Figure 3). Within these, 4 were fatty acids, including tetradecanedioic acid, stearidonic acid, palmitoylcarnitine, and acetylcarnitine.

Fatty acids are a major energy source for myocardial tissue, and long-chain fatty acids in particular have been implicated in cardiovascular events [27]. These reports are in line with previous evidence. In murine models of hypertension stearidonic acid was found to be significantly higher compared to normotensive controls [28]. Moreover, the alpha, omega-dicarboxylic acid, tetradecanedioic acid, is a close relative to hexadecanedioic acid, another long-chain dicarboxylic acid with causal evidence linked to increased BP [11].

Within the 14 positively associated lipids were also 2 hydroxy- and 1 androstane-steroids, namely, cortisol, cortisone and 5-androstenediol. Elevated levels of steroid hormones have been implicated with an increased risk for the development of hypertension [24]. Cushing’s syndrome, a disorder of over secretion of cortisol, presents with high BP [29], and this is believed to be a direct consequence of the effect of cortisol on adrenocorticotrophic hormone. Moreover, an infusion of cortisol over a 5-day period has been shown to increase SBP by 21 mmHg [30].

The current study has several strengths. By leveraging the COMETS our study is to-date the largest MWAS of hypertension, with a substantial sample size to detect any rare metabolites. This also facilitates the inclusion of cohorts spanning multiple continents with diverse ethnic demographics. The MWAS approach also facilitated the inclusion of a range of metabolomics platforms, increasing our scope for potential hypertension-associated metabolites.

However, despite these strengths our results must be appreciated in the presence of several limitations. Firstly, although we account for potential confounders including, dietary intake and comorbidities, in large prospective-observational cohorts, food frequency questionnaires are the typical method for dietary data collection, while comorbidities are recorded by health questionnaires, rather than derived from medical records. The self-reported nature of this data may introduce reporting bias, which includes social desirability and selective recall, resulting in misclassification. Such misclassification of confounders could result in residual confounding. Furthermore, cohorts were included in the sensitivity analyses even if they did not have data on all potential confounders, and this would also introduce residual confounding. Moreover, non-hypertensive controls, may have had other comorbidities, which could contribute to changes in metabolism and therefor a potential source of confounding. Second, given the study designs of the contributing cohorts data included may not have been recorded/reported on the same day. Third, despite leveraging the COMETS, our sample was not represented equally by all race/ethnicities, and was mostly comprised of White/European individuals, consequently our stratified analysis in those of Black ethnicity may not have been sufficiently powered, and we were unable to have an appreciable overlap of metabolites to stratify our sample by Asian/Hispanic or other ancestries. Moreover, in our stratified analysis not all metabolites were present in >1 cohort and >80% of the stratified sample, therefore comparisons between sexes and ethnicities for some metabolites cannot be made. Additionally, regional disparities in the metabolome have previously been reported [31], but we were unable to account for it, as specific regional data was not available. Fourth, to incorporate all cohorts, here we have used cross-sectional metabolomics. This lack of longitudinal metabolomics prevents us from investigating causality or validating the predictive power of these metabolites in relation to hypertension. Moreover, hypertension was defined from a single timepoint, and we were unable to describe the potential duration of hypertension, as longitudinal BP data was unavailable in all cohorts. Lastly, although we include over 44,000 individuals and multiple metabolomic platforms, metabolites are not measured by each platform, thus the limited overlap between platforms reduces statistical power.

## 4. Methods

A flowchart of the study design is presented in Supplementary Figure S1.

### 4.1. Study Populations

This study is drawn from COMETS, which is described in detail elsewhere [16].

Here we included cohorts from COMETS that had concurrent systolic blood pressure (SBP), diastolic blood pressure (DBP), and metabolomics on participants without pre-determined renal impairment. No a priori exclusion was applied to cohorts for the number of hypertensive cases (mean cases = 1387). Additional requested covariates included: age, sex, BMI, ethnicity, use of antihypertensive medication, co-morbidities (prevalence of cancer, diabetes, and heart disease), serum creatinine, and dietary information (salt and alcohol intake). We also included data from the Qatar Biobank cohort, a non-COMETS member, but with data matching the COMETS recruitment criteria (Supplementary Figure S1).

A total of nine prospective cohorts were recruited spanning three continents, and are described in Supplementary Table S1. They include Asia: Qatar Biobank (QBB), a population-based cohort study in Qatar, the first of its kind in the Gulf region [32]; Europe: Avon Longitudinal Study of Parents and Children (ALSPAC), a multi-generation birth cohort study, recruited from the former county of Avon in South-West England [33,34,35,36], Born in Bradford (BIB), a multi-ethnic pregnancy and birth cohort recruited from the north of England [37], Caerphilly Prospective Study (CaPS), an epidemiological cohort with a sample representative of a small town in South Wales, UK [38], European Prospective Investigation into Cancer and Nutrition (EPIC), a multi-centre cohort comprised of 23 centres in 10 European countries [39], TwinsUK, the largest cohort of community-dwelling adult twins in the UK [40], and Whitehall II (WII), a longitudinal cohort of British civil servants recruited from London, England [41]; North America: Atherosclerosis Risk in Communities Study (ARIC), a large prospective study recruited from four US communities of Minnesota, North Carolina, Maryland, and Mississippi [42], and Health, Aging and Body Composition Study (HealthABC), a mixed ethnicity cohort recruited from four US states, including Memphis, Tennessee or Pittsburgh, and Pennsylvania [43] (Supplementary Figure S1).

### 4.2. Phenotypes

#### 4.2.1. Metabolomics

Metabolites were quantified from blood in each study using targeted or untargeted assays (Supplementary Table S1), with most studies utilising mass-spectrometry from Metabolon, Inc. (Morrisville, NC, USA). Metabolites were aligned between studies and across platforms using the universal ID developed by the COMETS harmonisation working group [44], and where not available by the Human Metabolome Database [45] (HMDB) identifier directly from each cohort.

#### 4.2.2. Blood Pressure

Both SBP and DBP were measured within each study (mmHg) from a single timepoint, at the closest visit to the metabolomics blood sample collection. Essential hypertension was then defined following the European Society of Hypertension 2021 guidelines [1]. If participants’ SBP ≥ 140 or their DBP ≥ 90, or they were using anti-hypertensive medication at the time of the BP measurement, they were classified as hypertensive cases, otherwise they were considered as non-hypertensive controls.

#### 4.2.3. Covariates

Within each study, demographics, lifestyle factors (diet), anthropometrics, and medical data were collected at the closest visit to the metabolomics blood sample. Variables included in this study were age; sex; ethnicity; BMI (kg/m^2^); use of antihypertensive medication; presence of cancer, diabetes, or heart disease; serum creatinine (µmol/L); dietary salt (g/day) and alcohol intake (g/day).

### 4.3. Statistical Analysis

Standardised pipelines were followed by each cohort to analyse data using the R programming language [46].

Ethnicity was defined using 4 levels: White/European, Black, Asian or Hispanic, or other ancestry. Salt intake was defined by a binary boundary, based on current recommendations [47], ≥6 g/day, or ≤6 g/day. Alcohol intake was categorised using 4 cut-offs, 0 g per day, >0 g and <15 g per day, ≥15 g per day and <30 g per day, or ≥30 g day.

For the current analyses, cohorts excluded all individuals predefined as renally impaired. To further mitigate potential confounding effects brought about by renal dysfunction, where serum creatinine was available, we estimated glomerular filtration rate (eGFR) using the Modification of Diet in Renal Disease equation [48], and individuals with an eGFR <60 were excluded. To account for inter-study differences and improve normality all metabolites were quantile normalised using a rank-based inverse normal transformation [49], and to mitigate spurious associations metabolites were excluded if present in <80% of the sample.

Logistic regressions were used to estimate the associations between metabolites and hypertension status. Covariates were determined a priori to the analysis based on previous literature. Basic models were adjusted for traditional risk factors (age, age^2^, sex, BMI, and ethnicity) and, where necessary, batch correction was included. As a sensitivity analysis we ran a second multivariable-adjusted model further adjusted for dietary salt and alcohol intakes and the prevalence of comorbidities (dichotomous variables for cancer, diabetes, and heart disease,). As a sub-analysis, we also ran both models stratified by (i) sex and (ii) ethnicity.

As we wanted to include fully annotated and therefore actionable metabolites, here we only included metabolites that were identifiable by a HMDB id and were analysed in more than 1 cohort. First, we ran random-effects inverse variance meta-analyses using the R package “meta” to pool estimates from the basic model. *p* values were corrected for multiple testing using a Bonferroni correction (0.05 × 813 = 6.15 × 10^−5^). For all hypertension-associated metabolites (after multiple testing) we tested the robustness of associations by further pooling the estimates for the multivariable adjusted model using the same methods (Supplementary Figure S1). Heterogeneity in study-specific estimates were examined using the Cochrane Q-value and I^2^ statistics.

We then conducted a canonical pathway analysis using the Ingenuity pathway analysis (IPA) database [50] to investigate metabolic pathways underlying the hypertension-associated metabolites (Supplementary Figure S1). A right-tailed Fisher’s exact test was used to calculate a *p*-value determining the probability (α = 0.05) that the association between the hypertension-associated metabolites and the canonical pathway was not explained by chance alone.

## 5. Conclusions

In the largest metabolome wide association study of hypertension to date, including multi-ethnic cohorts, we report 5 novel hypertension-associated metabolites and confirm 27 previous hypertension-associations, highlighting the influence of amino acids, and lipids—including the novel positive-relationship with bile acids. Canonical pathway analysis supports these findings. The clinical implications of these metabolites lie in a series of follow-up studies investigating the molecular pathways related to hypertension, as this may lead to the identification of molecular mechanisms involved in cardiovascular diseases, particularly those linked to glycine, serine, and bile acids, that act through other pathways. The identification of key metabolites related to hypertension should encourage further research into this field.

## Figures and Tables

**Table 1:** Descriptive characteristics of included cohorts.

Cohort	ALSPAC	ARIC	BIB	CaPS	EPIC	HealthABC	QBB	TwinsUK	Whitehall II
*n*	9396	3293	1795	989	16,418	232	2906	4427	4850
	mean (SD)	mean (SD)	mean (SD)	mean (SD)	mean (SD)	mean (SD)	mean (SD)	mean (SD)	mean (SD)
Age	40.9 (12.5)	52.9 (5.5)	28 (5.7)	61.4 (4.4)	56.1 (8)	74.7 (2.8)	39 (12)	54 (13.3)	56.2 (6)
BMI	26.1 (5)	28.9 (5.9)	26.8 (5.9)	26.8 (3.7)	26.7 (4.1)	27.1 (4.4)	28.9 (5.9)	26.1 (4.8)	26.3 (3.9)
SBP	120.3 (13.8)	129.8 (23.3)	110.6 (11.8)	148.6 (24.4)	138.3 (22.5)	138.1 (23.3)	115.9 (17)	127.4 (18.6)	125.2 (18.3)
DBP	71.1 (9.1)	79.9 (13.5)	65.6 (8.5)	84.1 (13.2)	84.8 (12.6)	77.9 (14.3)	74 (11.2)	78.5 (10.8)	78.6 (11.3)
	*n* (%)	*n* (%)	*n* (%)	*n* (%)	*n* (%)	*n* (%)	*n* (%)	*n* (%)	*n* (%)
HTN cases	1007 (10.7)	1501 (45.6)	18 (1)	631 (63.8)	6897 (42)	48 (20.7)	409 (14.1)	1322 (29.9)	646 (13.3)
Non-HTN controls	8389 (89.3)	1792 (54.4)	1777 (99)	358 (36.2)	9521 (58)	184 (79.3)	2497 (85.9)	3105 (70.1)	4204 (86.7)
Sex									
Males	3062 (33)	1375 (41.8)	0	989 (100)	8659 (52.7)	232 (100)	1457 (50.1)	339 (7.7)	3329 (68.6)
Females	6334 (67)	1918 (58.2)	1795 (100)	0	7759 (47.3)	0	1449 (49.9)	4088 (92.3)	1521 (31.4)
Ancestry									
White	8436 (89.8)	1053 (32)	842 (46.9)	989 (100)	NA	0	0	3653 (82.5)	4475 (92.3)
Black	40 (0.4)	2240 (68)	0	0	NA	232 (100)	0	16 (0.4)	103 (2.1)
Asian/Hispanic	43 (0.5)	0	953 (53.1)	0	NA	0	2906 (100)	27 (0.6)	234 (4.8)
Other	37 (0.4)	0	0	0	NA	0	0	0	37 (0.7)

Abbreviations: SD, standard deviation; ALSPAC, Avon Longitudinal Study of Parents and Children; ARIC, Atherosclerosis Risk in Communities Study; BIB, Born in Bradford; CaPS, Caerphilly Prospective Study; EPIC, European Prospective Investigation into Cancer and Nutrition; HealthABC, Health, Aging and Body Composition Study; QBB, Qatar Biobank; BMI, body mass index; SBP, systolic blood pressure; DBP diastolic blood pressure.

## Data Availability

The data used in this study are held by the department of Twin Research at King’s College London. The data relating to TwinsUK can be released to bona fide researchers using our normal procedures overseen by the Wellcome Trust and its guidelines as part of our core funding (https://twinsuk.ac.uk/resources-for-researchers/access-our-data/, accessed on 8 June 2022). However, data relating to the Qatar Biobank, Avon Longitudinal Study of Parents and Children, Born in Bradford, Caerphilly Prospective Study, Whitehall II, Atherosclerosis Risk in Communities Study, Health, Aging and Body Composition Study, will require additional approval steps.

## Supplementary Materials

The following supporting information can be downloaded at: https://www.mdpi.com/article/10.3390/metabo12070601/s1, Supplementary Figure S1: Flowchart of study analytical pipeline.; Supplementary Figure S2: Heatmap of overall and stratified analyses grouped by metabolite class; Supplementary Table S1: Descriptive table of contributing COHORTS; Supplementary Table S2: Hypertension-associated metabolites. References [51,52,53,54,55,56,57,58,59,60,61,62] are cited in the Supplementary Materials.
