# Supplementary material for: Cross-Sectional Blood Metabolite Markers of Hypertension: A Multicohort Analysis of 44,306 Individuals from the COnsortium of METabolomics Studies
Source: Metabolites. 2022 Jun 28;12(7):601. doi: 10.3390/metabo12070601 (PMC9324896; doi:10.3390/metabo12070601)
Supplement: Supplementary file 1 [file metabolites-12-00601-s001.zip › metabolites-1751404-Supplementary.pdf]

Supplementary Table S1. Descriptive table of contributing COHORTS.

Study Name	Cohort abbreviation	Location	Platform or lab	Analytical technology	Targeted/untargeted	Description
**Avon Longitudinal Study of Parents and Children**	ALSPAC	Europe	Nightingale Health	NMR	Targeted	Pregnant women resident in Avon, UK with expected dates of delivery 1st April 1991 to 31st December 1992 were invited to take part in the study. The initial number of pregnancies enrolled is 14,541 (for these at least one questionnaire has been returned or a "Children in Focus" clinic had been attended by 19/07/99). Of these initial pregnancies, there was a total of 14,676 foetuses, resulting in 14,062 live births and 13,988 children who were alive at 1 year of age.<br>When the oldest children were approximately 7 years of age, an attempt was made to bolster the initial sample with eligible cases who had failed to join the study originally. As a result, when considering variables collected from the age of seven onwards (and potentially abstracted from obstetric notes) there are data available for more than the 14,541 pregnancies mentioned above. The number of new pregnancies not in the initial sample (known as Phase I enrolment) that are currently represented on the built files and reflecting enrolment status at the age of 24 is 913 (456, 262 and 195 recruited during Phases II, III and IV respectively), resulting in an additional 913 children being enrolled. The phases of enrolment are described in more detail in the cohort profile paper and its update [1-3]. The total sample size for analyses using any data collected after the age of seven is therefore 15,454 pregnancies, resulting in 15,589 foetuses. Of these 14,901 were alive at 1 year of age.<br>A 10% sample of the ALSPAC cohort, known as the Children in Focus (CiF) group, attended clinics at the University of Bristol at various time intervals between 4 to 61 months of age. The CiF group were chosen at random from the last 6 months of ALSPAC births (1432 families attended at least one clinic). Excluded were

those mothers who had moved out of the area or were lost to follow-up, and those partaking in another study of infant development in Avon.
For this project we included eligible mothers, fathers/partners, and children (at approximately age 24). Study data were collected and managed using Research Electronic Data Capture (REDCap) electronic data capture tools hosted at the University of Bristol [4]. REDCap is a secure, web-based software platform designed to support data capture for research studies. Please note that the study website contains details of all the data that is available through a fully searchable data dictionary and variable search tool (http://www.bristol.ac.uk/alspac/researchers/our-data/).
Ethical approval for the study was obtained from the ALSPAC Ethics and Law Committee and the Local Research Ethics Committees.
We are extremely grateful to all the families who took part in this study, the midwives for their help in recruiting them, and the whole ALSPAC team, which includes interviewers, computer and laboratory technicians, clerical workers, research scientists, volunteers, managers, receptionists, and nurses.
The UK Medical Research Council and Wellcome (Grant ref: 217065/Z/19/Z) and the University of Bristol provide core support for ALSPAC. Deborah Lawlor, and Neil Goulding contributed to this work and will serve as guarantors for the ALSPAC contents of this paper. A comprehensive list of grants funding is available on the ALSPAC website (http://www.bristol.ac.uk/alspac/external/documents/grant-acknowledgements.pdf). Work from ALSPAC authors was specifically funded by the British Heart Foundation (Grant ref: SP/07/008/24066 and CS/15/6/31468), Wellcome Trust (Grant ref: WT092830/Z/10/ Z), Wellcome Trust and MRC (Grant ref: 092731) and NIHR (Grant ref: NF-SI-0611-10196).

**Atherosclerosis Risk in Communities Study**	ARIC	North America	Metabolon	GC-MS, LC-MS	Untargeted	Prospective cohort recruited from 4 U.S communities to investigate the aetiology of atherosclerosis and its clinical outcomes.
**Born in Bradford**	BIB	Europe	Metabolon	GC-MS, LC-MS	Untargeted	The Born in Bradford (BiB) study is a population-based prospective UK longitudinal birth cohort. 12,453 women who experienced 13,776 pregnancies, were recruited at their oral glucose tolerance test (OGTT) at approximately 26–28 weeks' gestation, which was offered to all women booked for delivery at Bradford Royal Infirmary. Eligible women had an expected delivery between March 2007 and December 2010. The study is unique because it includes high proportions of White European and South Asian families, all residing in Bradford, UK. Bradford is a city in the North of England with high levels of socio- economic deprivation, and the cohort was started due to a high prevalence of poor child health in the city [5]. Mothers recruited into the study provided detailed questionnaire data, measurements, and biological samples at baseline (OGTT) and after birth. Infants have had detailed anthropometric assessment at birth and postnatally. Both targeted and untargeted mass-spectrometry (MS) metabolite data have been obtained from the cohort. For this project, we included eligible mothers from the metabolites dataset which had the greatest overlap of metabolites with the other cohorts; this was the BiB 2000 MS dataset, which is a subgroup selected using a case cohort design [6].<br>All study participants gave written informed consent and ethical approval was obtained from the Bradford Research Ethics Committee.
**Caerphilly Prospective Study**	CaPS	Europe	Nightingale Health	NMR	Targeted	Prospective study established in 1979 to examine the importance of lipids, haemostatic factors, and hormones (testosterone, cortisol, insulin etc.) in the development of ischaemic heart disease.
**Copenhagen Prospective**	COPSAC	Europe	Metabolon	LC-MS	Untargeted	Population-based mother-child cohort in which 700 pregnant women were recruited between 22 and 26 weeks of gestation and

**Studies on Asthma in Childhood**						their children subsequently followed prospectively during their first five years of life at 12 scheduled clinical visits.
**European Prospective Investigation into Cancer and Nutrition**	EPIC	Europe	IARC	LC-MS	Targeted	A cohort recruited from 10 European countries designed to explore the relationship between diet, lifestyle and environmental factors and incidence of cancer.
**Health, Aging and Body Composition**	HealthABC	North America	Broad Institute	LC-MS	Untargeted	Longitudinal cohort designed to investigate differences in frailty and longevity.
**Qatar Biobank**	QBB	Asia	Metabolon	LC-MS	Untargeted	Prospective, population-based cohort study in Qatar, established to investigate a host of health-related questions through evidence-based research.
**TwinsUK**	-	Europe	Metabolon	GC-MS, LC-MS	Untargeted	The largest most clinically characterised adult twin registry in the UK, recruited as volunteers without selecting for particular diseases or traits.
**Stress and Health Study**	Whitehall II	Europe	Nightingale Health	LC-MS	Targeted	Prospective cohort established to investigate the causes of social inequalities in health.

Supplementary Table S2. Hypertension-associated metabolites.

Taxonomy				Sensitivity analysis	Previously reported (HTN/BP)
**Super class**	**Class**	**Sub Class**	**Metabolite**		
Lipids	Steroids and steroid derivatives	Hydroxysteroids	Cortisol	✓	7
			Cortisone	✓	7
		Bile acids, alcohols	Glycocholic acid	✓	
			Chenodeoxycholic acid glycine conjugate	✓	
		Androstane steroids	5-Androstenediol	✓	8
	Fatty Acyls	Fatty acid esters	L-Acetylcarnitine	✓	7
			L-Palmitoylcarnitine	✓	7
			Butyrylcarnitine	×	9
		Fatty acids and conjugates	2-Hydroxy-3-methylbutyric acid	×	
			Tetradecanedioic acid	✓	10
		Lineolic acids	Stearidonic acid	✓	11
	Glycerophospholipids	Glycerophosphoethanolamines	LysoPE(16:0/0:0)	✓	12
			LysoPE(20:4(5Z,8Z,11Z,14Z)/0:0)	✓	
			Glycerylphosphorylethanolamine	✓	
Organic Acids	Carboxylic acids	Amino acids, peptides, and analogues	Homocitrulline	✓	13
			L-Threonine	×	14
			Glycine	✓	15
			L-Isoleucine	✓	16
			L-Leucine	✓	16
			L-Valine	×	16
			L-Histidine	✓	17
			L-Serine	✓	15
			N-Acetyl-L-alanine	✓	18
	Hydroxy acids and derivatives	Alpha hydroxy acids	L-Lactic acid	✓	7
		Beta hydroxy acids	L-Malic acid	✓	7
	Keto acids and derivatives	Short-chain keto acids	Ketoleucine	✓	
			3-Methyl-2-oxovaleric acid	✓	7
Organic oxygen compounds	Organooxygen compounds	Alcohols and polyols	myo-Inositol	✓	11
			Pantothenic acid	✓	19
		Carbohydrates	Glycerol	✓	7
			Erythritol	✓	7
			Gluconic acid	×	20
Organoheterocyclic compounds	Imidazopyrimidines	Purines	Theophylline	✓	7
			Uric acid	×	7
			Caffeine	✓	7
	Pyridines and derivatives	Pyridoxines	Pyridoxine	✓	21

	Indoles and derivatives	Indolyl carboxylic acids	Indolelactic acid	✓	22
Phenylpropanoids and polyketides	Phenylpropanoic acids	N/A	Hydrocinnamic acid	✓	23

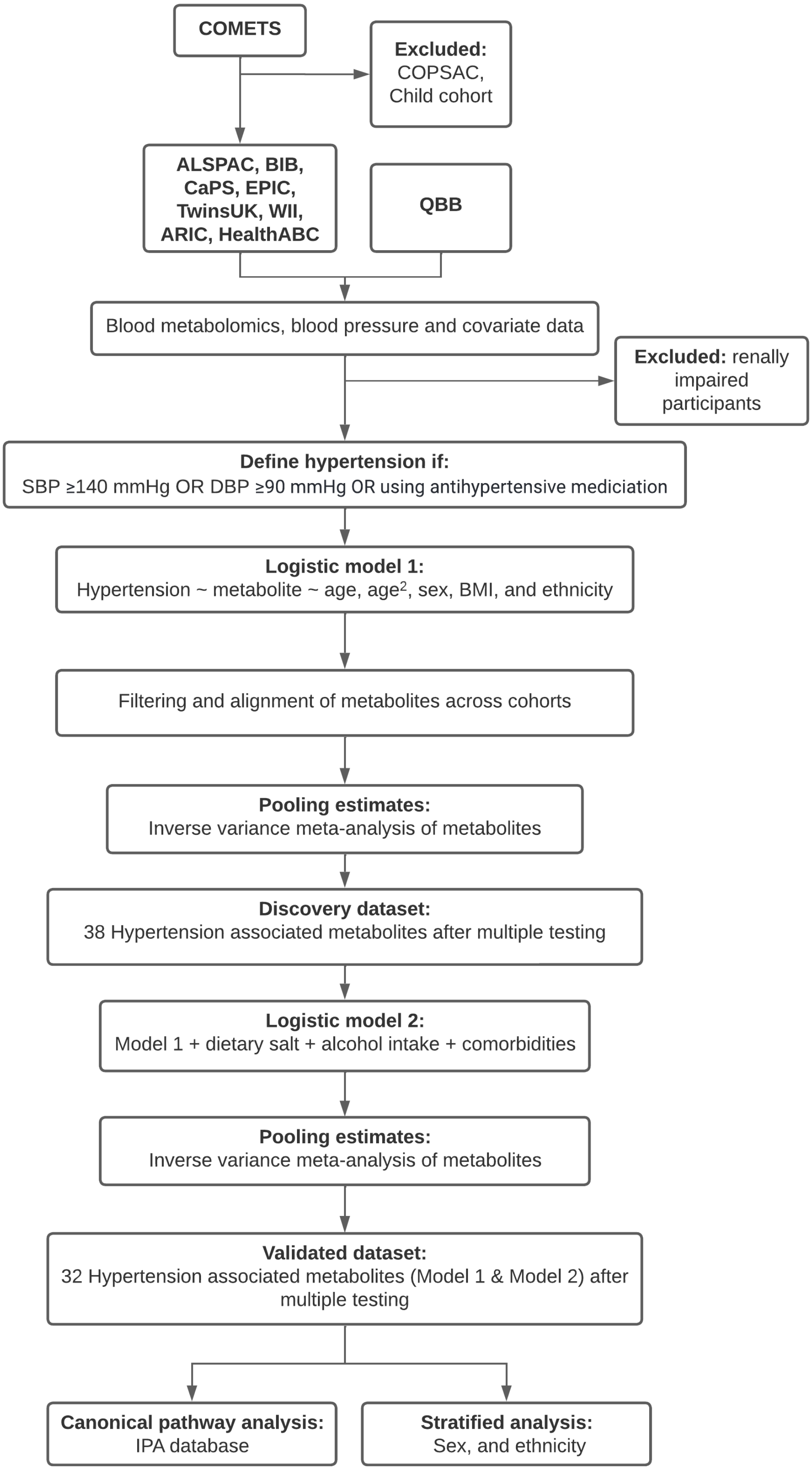

Supplementary Figure S1. Flowchart of study analytical pipeline.

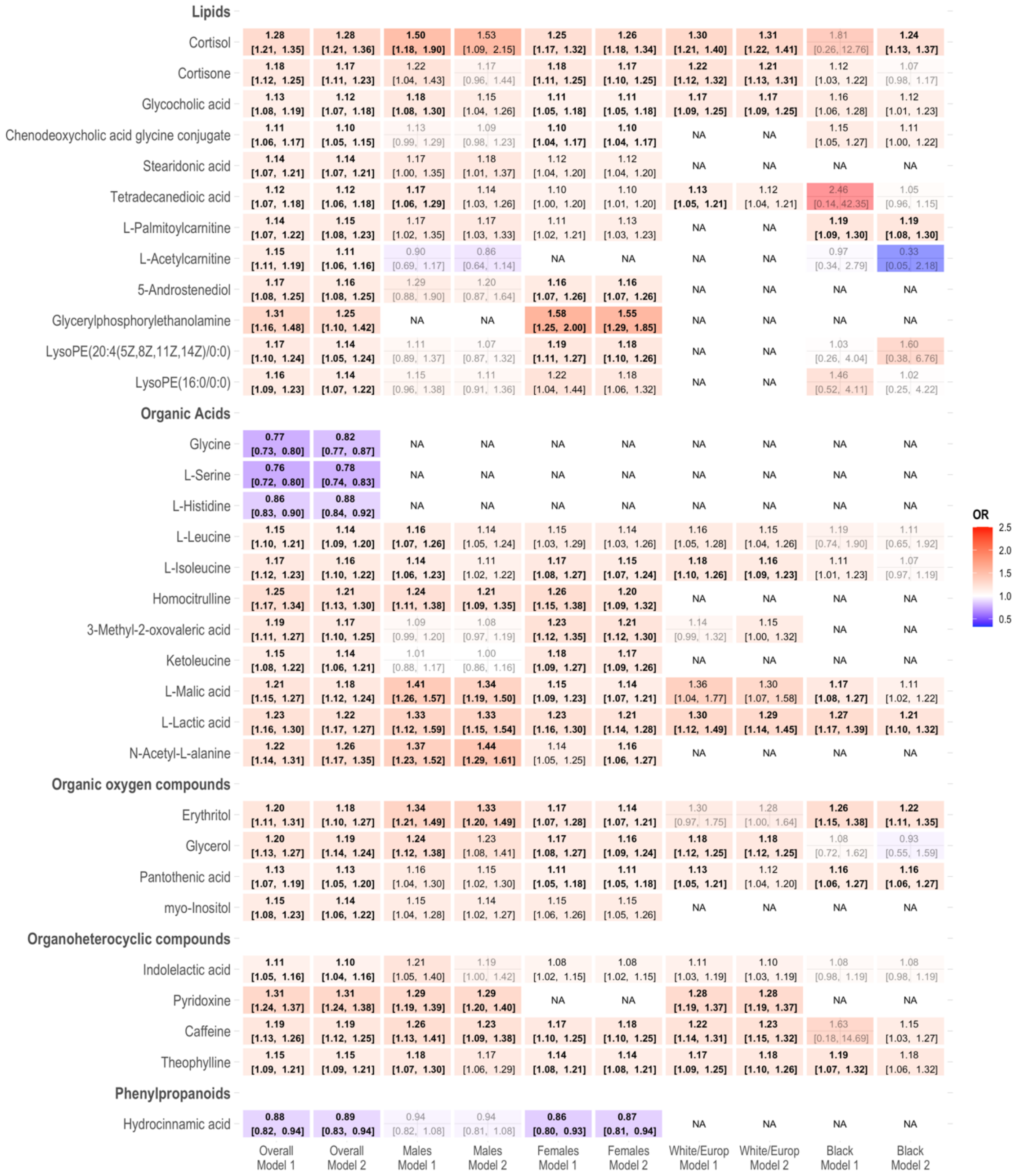

Supplementary Figure S2. Heatmap of overall and stratified analyses grouped by metabolite class. Colour scale of boxes represent the direction of effect (Odds ratio), those passing multiple testing are depicted in bold font, nominal significance in roman font, and non-significant values are semitransparent. Metabolites where there were fewer than 2 cohorts with the metabolite detected in >80% of the stratified sample are shown as NA.

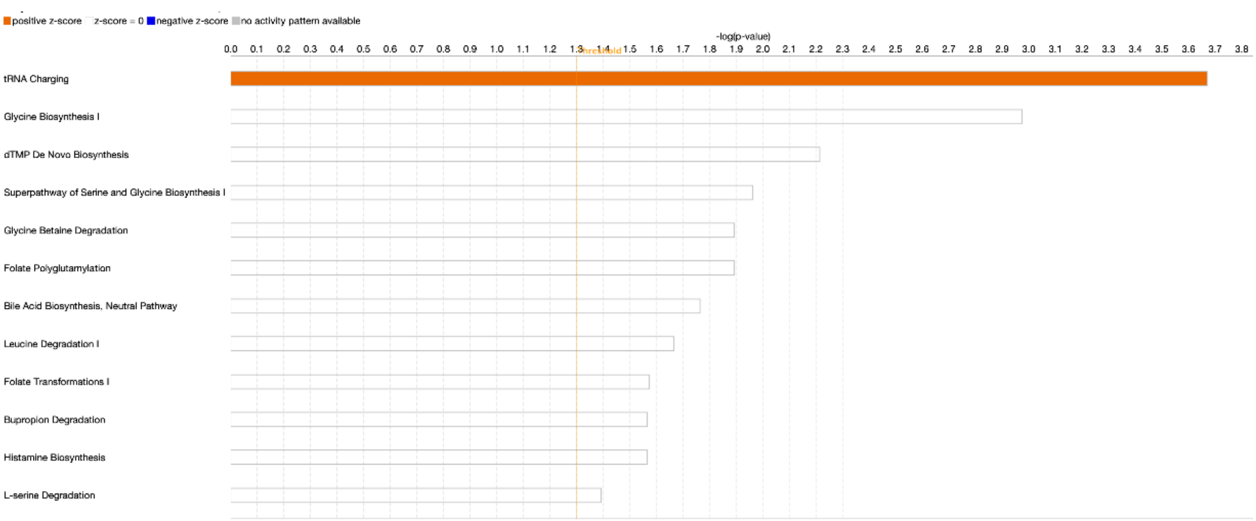

Supplementary Figure S3. Canonical pathway analysis of the 32 hypertension-associated metabolites in the IPA database. Bars represent the -log(p-value) for the Fisher's Exact test, colours of the bars represent the direction of expression based upon the effect sizes and direction of each of the metabolites.

References

1. Boyd, A.; Golding, J.; Macleod, J.; Lawlor, D.A.; Fraser, A.; Henderson, J.; Molloy, L.; Ness, A.; Ring, S.; Davey Smith, G. Cohort Profile: The 'Children of the 90s'—The index offspring of the Avon Longitudinal Study of Parents and Children. *Int. J. Epidemiol.* **2012**, *42*, 111–127. https://doi.org/10.1093/ije/dys064.
2. Fraser, A.; Macdonald-Wallis, C.; Tilling, K.; Boyd, A.; Golding, J.; Davey Smith, G.; Henderson, J.; Macleod, J.; Molloy, L.; Ness, A.; et al. Cohort Profile: The Avon Longitudinal Study of Parents and Children: ALSPAC mothers cohort. *Int. J. Epidemiol.* **2012**, *42*, 97–110. https://doi.org/10.1093/ije/dys066.
3. Northstone, K.; Lewcock, M.; Groom, A.; Boyd, A.; Macleod, J.; Timpson, N.; Wells, N. The Avon Longitudinal Study of Parents and Children (ALSPAC): An update on the enrolled sample of index children in 2019 [version 1; peer review: 2 approved]. *Wellcome Open Res.* **2019**, *4*, 51. https://doi.org/10.12688/wellcomeopenres.15132.1.
4. Harris, P.A.; Taylor, R.; Thielke, R.; Payne, J.; Gonzalez, N.; Conde, J.G. Research electronic data capture (REDCap)—A metadata-driven methodology and workflow process for providing translational research informatics support. *J. Biomed. Inform.* **2009**, *42*, 377–381. https://doi.org/10.1016/j.jbi.2008.08.010.
5. Wright, J.; Small, N.; Raynor, P.; Tuffnell, D.; Bhopal, R.; Cameron, N.; Fairley, L.; Lawlor, D.A.; Parslow, R.; Petherick, E.S.; et al. Cohort Profile: The Born in Bradford multi-ethnic family cohort study. *Int. J. Epidemiol.* **2012**, *42*, 978–991. https://doi.org/10.1093/ije/dys112.
6. Taylor, K.; McBride, N.; JGoulding, N.; Burrows, K.; Mason, D.; Pembrey, L.; Yang, T.; Azad, R.; Wright, J.; ALawlor, D. Metabolomics datasets in the Born in Bradford cohort [version 2; peer review: 1 approved, 1 approved with reservations]. *Wellcome Open Res.* **2021**, *5*, 264.
7. Menni, C.; Graham, D.; Kastenmüller, G.; Alharbi, N.H.; Alsanosi, S.M.; McBride, M.; Mangino, M.; Titcombe, P.; Shin, S.Y.; Psatha, M.; et al. Metabolomic identification of a novel pathway of blood pressure regulation involving hexadecanedioate. *Hypertension* **2015**, *66*, 422–9.
8. Hughes, G.S.; Mathur, R.S.; Margolius, H.S. Sex steroid hormones are altered in essential hypertension. *J. Hypertens.* **1989**, *7*, 181–187.
9. Goïta, Y.; Chao de la Barca, J.M.; Keïta, A.; Diarra, M.B.; Dembélé, K.C.; Chabrun, F.; Dramé, B.S.I.; Kassogué, Y.; Diakité, M.; Mirebeau-Prunier, D.; et al. Sexual Dimorphism of Metabolomic Profile in Arterial Hypertension. *Sci. Rep.* **2020**, *10*, 7517.
10. Li, L.; Zhong, S.; Hu, S.; Cheng, B.; Qiu, H.; Hu, Z. Changes of gut microbiome composition and metabolites associated with hypertensive heart failure rats. *BMC Microbiol.* **2021**, *21*, 141.
11. Lu, Y.; Wang, G.; Hao, H.; Huang, Q.; Yan, B.; Zha, W.; Gu, S.; Ren, H.; Zhang, Y.; Fan, X.; et al. Gas chromatography/time-of-flight mass spectrometry based metabonomic approach to differentiating hypertension- and age-related metabolic variation in spontaneously hypertensive rats. *Rapid Commun. Mass Spectrom.* **2008**, *22*, 2882–2888. https://doi.org/10.1002/rcm.3670.
12. Li, J.; Zhao, F.; Wang, Y.; Chen, J.; Tao, J.; Tian, G.; Wu, S.; Liu, W.; Cui, Q.; Geng, B.; et al. Gut microbiota dysbiosis contributes to the development of hypertension. *Microbiome* **2017**, *5*, 14.

13. Lu, J.; Tian, Y.; Gu, J.; Qiu, M.; Lu, Y.; Sun, W.; Kong, X. Comparative Study of Metabolite Changes after Antihypertensive Therapy with Calcium Channel Blockers or Angiotensin Type 1 Receptor Blockers. *J. Cardiovasc. Pharmacol.* **2021**, *77*, 228–237.
14. Hao, Y.; Wang, Y.; Xi, L.; Li, G.; Zhao, F.; Qi, Y.; Liu, J.; Zhao, D. A Nested Case-Control Study of Association between Metabolome and Hypertension Risk. *BioMed Res. Int.* **2016**, *2016*, 7646979.
15. Dietrich, S.; Floegel, A.; Weikert, C.; Prehn, C.; Adamski, J.; Pischon, T.; Boeing, H.; Drogan, D. Identification of Serum Metabolites Associated With Incident Hypertension in the European Prospective Investigation Into Cancer and Nutrition-Potsdam Study. *Hypertension* **2016**, *68*, 471–477. https://doi.org/10.1161/HYPERTENSIONAHA.116.07292.
16. Flores-Guerrero, J.L.; Groothof, D.; Connelly, M.A.; Otvos, J.D.; Bakker, S.J.L.; Dullaart, R.P.F. Concentration of Branched-Chain Amino Acids Is a Strong Risk Marker for Incident Hypertension. *Hypertension* **2019**, *74*, 1428–1435. https://doi.org/10.1161/HYPERTENSIONAHA.119.13735.
17. Toba, H.; Nakamori, A.; Tanaka, Y.; Yukiya, R.; Tatsuoka, K.; Narutaki, M.; Tokitaka, M.; Hariu, H.; Kobara, M.; Nakata, T. Oral L-histidine exerts antihypertensive effects via central histamine H3 receptors and decreases nitric oxide content in the rostral ventrolateral medulla in spontaneously hypertensive rats. *Clin. Exp. Pharm. Physiol.* **2010**, *37*, 62–8.
18. Zheng, Y.; Yu, B.; Alexander, D.; Manolio, T.A.; Aguilar, D.; Coresh, J.; Heiss, G.; Boerwinkle, E.; Nettleton, J.A. Associations between Metabolomic Compounds and Incident Heart Failure Among African Americans: The ARIC Study. *Am. J. Epidemiol.* **2013**, *178*, 534–542. https://doi.org/10.1093/aje/kwt004.
19. Schwabedal, P.E.; Pietrzik, K.; Wittkowski, W. Pantothenic Acid Deficiency as a Factor Contributing to the Development of Hypertension. *Cardiology* 1985, 72, (Suppl. S1), 187-189.
20. Ho, A.; Sinick, J.; Esko, T.; Fischer, K.; Menni, C.; Zierer, J.; Matey-Hernandez, M.; Fortney, K.; Morgen, E.K. Circulating glucuronic acid predicts healthspan and longevity in humans and mice. *Aging* **2019**, *11*, 7694–7706.
21. Fregly, M.J.; Cade, J.R. Effect of Pyridoxine and Tryptophan, Alone and Combined, on the Development of Deoxycorticosterone Acetate-Induced Hypertension in Rats. *Pharmacology* **1995**, *50*, 298–306.
22. Dzúrik, R.; Fekovská, N.; Brimichová, G.; Tiso, P. Blood Pressure, 5-OH Indoleacetic Acid, and Vanilmandelic Acid Excretion and Blood Platelet Aggregation in Hypertensive Patients Treated with Ketanserin. *J. Cardiovasc. Pharmacol.* **1985**, *7*, 29–31.
23. Alam, M.A. Anti-hypertensive Effect of Cereal Antioxidant Ferulic Acid and Its Mechanism of Action. *Front. Nutr.* **2019**, *6*, 121.
